# Classification of Level of Consciousness in a Neurological ICU Using Physiological Data

**DOI:** 10.1007/s12028-022-01586-0

**Published:** 2022-09-15

**Authors:** Louis A. Gomez, Qi Shen, Kevin Doyle, Athina Vrosgou, Angela Velazquez, Murad Megjhani, Shivani Ghoshal, David Roh, Sachin Agarwal, Soojin Park, Jan Claassen, Samantha Kleinberg

**Affiliations:** 1 Stevens Institute of Technology, 1 Castle Point on Hudson, Hoboken, NJ 07030, USA.; 2 Department of Neurology, Columbia University Irving Medical Center, New York, NY, USA.; 3 New York Presbyterian Hospital, New York, NY, USA.

**Keywords:** Consciousness, Physiologic monitoring, Neurological ICU, Subarachnoid hemorrhage, Intracerebral hemorrhage

## Abstract

**Background:**

Impaired consciousness is common in intensive care unit (ICU) patients, and an individual’s degree of consciousness is crucial to determining their care and prognosis. However, there are no methods that continuously monitor consciousness and alert clinicians to changes. We investigated the use of physiological signals collected in the ICU to classify levels of consciousness in critically ill patients.

**Methods:**

We studied 61 patients with subarachnoid hemorrhage (SAH) and 178 patients with intracerebral hemorrhage (ICH) from the neurological ICU at Columbia University Medical Center in a retrospective observational study of prospectively collected data. The level of consciousness was determined on the basis of neurological examination and mapped to comatose, vegetative state or unresponsive wakefulness syndrome (VS/UWS), minimally conscious minus state (MCS−), and command following. For each physiological signal, we extracted time-series features and performed classification using extreme gradient boosting on multiple clinically relevant tasks across subsets of physiological signals. We applied this approach independently on both SAH and ICH patient groups for three sets of variables: (1) a minimal set common to most hospital patients (e.g., heart rate), (2) variables available in most ICUs (e.g., body temperature), and (3) an extended set recorded mainly in neurological ICUs (absent for the ICH patient group; e.g., brain temperature).

**Results:**

On the commonly performed classification task of VS/UWS versus MCS−, we achieved an area under the receiver operating characteristic curve (AUROC) in the SAH patient group of 0.72 (sensitivity 82%, specificity 57%; 95% confidence interval [CI] 0.63–0.81) using the extended set, 0.69 (sensitivity 83%, specificity 51%; 95% CI 0.59–0.78) on the variable set available in most ICUs, and 0.69 (sensitivity 56%, specificity 78%; 95% CI 0.60–0.78) on the minimal set. In the ICH patient group, AUROC was 0.64 (sensitivity 56%, specificity 65%; 95% CI 0.55–0.74) using the minimal set and 0.61 (sensitivity 50%, specificity 80%; 95% CI 0.51–0.71) using the variables available in most ICUs.

**Conclusions:**

We find that physiological signals can be used to classify states of consciousness for patients in the ICU. Building on this with intraday assessments and increasing sensitivity and specificity may enable alarm systems that alert physicians to changes in consciousness and frequent monitoring of consciousness throughout the day, both of which may improve patient care and outcomes.

## Introduction

Treating intensive care unit (ICU) patients is challenging, as it requires high-stakes decisions to be made in complex and time-constrained environments [1, 2]. As part of patient care, the function of organ systems are monitored to guide treatments and interventions that must be administered quickly. In current clinical practice, several techniques and systems are already in place that continuously monitor function in organs such as the lungs [3, 4] and the heart [5–7]. To monitor brain function, however, clinicians rely on behavioral assessments. Some examples of these assessments are the Glasgow Coma Scale (GCS) [8], the Full Outline of Unresponsiveness (FOUR) [9], command following scale [10], and the Coma Recovery Scale–Revised (CRS-R) [11]. The most accurate of these assessments, the CRS-R, is performed infrequently (typically once a day); when it is used, it is time consuming and thus does not provide the regular insights available for other aspects of patient physiology. Further, repeated assessments within a short time frame are needed to reduce misdiagnosis because daily assessments provide only a brief window into consciousness, which can fluctuate throughout the day [12, 13]. Prior work found that such fluctuations are associated with worse outcomes (death, disability) at 3 months after subarachnoid hemorrhage (SAH) [14]. Thus, a patient’s state of consciousness is a major factor that guides patient care, intervention, and prognosis [8].

Given the difficulty involved in assessing consciousness using behavioral methods [15], alternative approaches have been explored. Electroencephalogram (EEG) allows for continuous measurement at the bedside, and previous research has shown that bedside EEG features correlate with the level of consciousness in a population with SAH [10]. However, continuous bedside EEG is only available in a select number of ICUs, and the measures investigated, to date, are still experimental. Other forms of brain monitoring such as functional EEG (fEEG) and functional magnetic resonance imaging (fMRI) have been used to test for consciousness directly by using mental (motor or spatial) imagery tasks [16–19] or local–global paradigms [20, 21], but these require patient participation, can only be performed intermittently, and are experimental and thus are not well suited to serve as alarm triggers. Motion sensing, by using wearable devices placed on all extremities, has been investigated for detecting neurological states but performs best on patients who are less impaired and requires additional sensors beyond those that are used clinically [22].

Given the limitations of prior methods for assessing consciousness, there is a critical need to develop tools to provide more frequent assessments of consciousness. Although physiological signals have not been extensively examined as a means to continually monitor consciousness, prior work has shown that time lags between physiological signals may be correlated with states of consciousness [23]. That work showed that the time lag between correlated variables (such as intracranial pressure [ICP] and brain oxygenation) was delayed in patients with SAH who had impaired consciousness compared with those with intact consciousness. In this work, we leverage the large volumes of physiological data collected in the ICU and test the hypothesis that physiologic measures routinely collected in the hospital setting closely track behavioral assessments and can be used to classify states of consciousness. Although we begin by showing such signals can be used to classify daily behavioral assessments, ultimately, our use of routinely collected physiological data may enable continuous insights into states of consciousness between assessments.

## Methods

### Dataset

We used data collected prospectively from all patients with poor-grade aneurysmal SAH who underwent invasive brain monitoring and were admitted between 2006 and 2013 and all patients with spontaneous non-traumatic intracerebral hemorrhage (ICH) who were admitted between 2009 and 2017 to the neurological ICU (neuro-ICU) at Columbia University Medical Center. The data consist of physiological signals that are continuously recorded during each patient’s ICU stay, along with daily neurological examinations that assess each patient’s degree of consciousness. The study design was that of a retrospective analysis of prospectively collected data. Patients were included if (1) they had physiological signals recorded and (2) they had behavioral assessments performed and recorded. Patients were excluded if (1) they were under 18 years of age, (2) they were pregnant, or (3) they or their family did not consent to participate in the study. Patients provided informed consent when they were able to do so. Otherwise, a health care proxy or legally authorized representative did so. When there was no designated health care proxy or legally authorized representative and the patient was unable to provide informed consent, they were enrolled under a waiver of consent as long as a family member did not object. If a patient regained consciousness later, they provided consent or declined participation. The data used were collected as part of a study approved by the Columbia University Institutional Review Board (approval numbers AAA5384 and AAAD4775).

#### Physiological Signals

The continuously recorded signals include respiratory signals (respiratory rate [RR], end-tidal carbon dioxide [CO2EX], and blood oxygen level [SPO2%]), cardiovascular signals (heart rate [HR] and mean arterial pressure [MAP]), brain signals (brain temperature [BrT], ICP, and brain tissue oxygenation [PbtO2]), and body temperature (TMP). All physiological signals were recorded at a sampling frequency of 5 s by using a high-resolution acquisition system (BedmasterEX; Excel Medical Electronics Inc) from General Electric Solar 8000i monitors that was inserted into a Microsoft SQL database (see Supplemental Table 3, Supplemental file 2 for monitoring devices for all physiological signals) [24, 25]. Because of differences in etiology, treatment plans, and monitoring, each patient may have different subsets of these variables recorded at varying times throughout their ICU stay, leading to missing variables across patients and missing instances within variables (see Supplemental Table 4, Supplemental file 2 for missing data percentages).

#### Behavioral Assessments

Daily neurological examinations with behavioral assessments of consciousness were performed during morning rounds between 8 a.m. and 11 a.m. by attending physicians with sedation removed about an hour before each examination (when possible). The assessment scores were assigned retrospectively via chart review of the neurological examination and were validated in a prior study that examined the utility of bedside EEG features for predicting behavioral states [10]. The behavioral assessment mapped consciousness into six behavioral states: comatose, 0 (no response to stimulation); arousable to noxious stimuli, 1 (eyes open); arousable to verbal stimuli, 2; spontaneous eye-opening, 3; follows simple (one and two-step) commands, 4; and follows complex (three-step contralateral localization) commands, 5. We further group the scores into clinically relevant categories: comatose (coma), 0; vegetative state (VS) or unresponsive wakefulness syndrome (UWS), 1; minimally conscious state minus (MCS−), 2 or 3; and command following (CF), 4 or 5. Additionally, we create a noncommand following (non-CF) group with the coma, UWS/VS, and MCS− categories.

### Data Processing

To preprocess the physiological signals, we first resampled the signals to nonoverlapping intervals of 1 min (consistent with prior work on this dataset [23, 24]), imputed missing data within each signal by using the Fourier Lagged *k*-nearest neighbors imputation [26], and then filtered outliers from each signal based on clinically recommended ranges (as in prior work on this dataset [24, 25]; see Supplemental Table 3, Supplemental file 2, for the filter ranges to remove outliers for each physiological signal). Note that imputation is performed separately for each patient, and we do not impute signals that are completely missing (see Supplemental file 1). Sedation is usually removed about an hour before each neurological examination, so we use physiological signals recorded before that time. We do this because most ICU patients are sedated, and using physiological signals recorded after removal of sedation would limit when our model can be used for classification. We use physiological signals from 60 to 200 min before an assessment because this window is large enough for feature extraction. We preliminarily tested window sizes of 1 to 3 h (starting at 60 min prior to assessment) on the task of VS/UWS versus MCS− task (using the proposed classification pipeline) and found 60–200 to perform best. If all variables were missing for more than 80% of that time window before an assessment, we excluded that assessment from the study (see Supplemental Table 4 in Supplemental file 2, which gives the percentages of variables missing after extracting time windows across all assessments). All code used for data processing and experiments is shared in our GitHub repository: https://github.com/health-ai-lab/consciousness-classification.

### Feature Extraction

After filtering out outliers from each signal, we then removed high-frequency noise from each signal in the extracted time window using a discrete wavelet transform (DWT) (level 2 DWT with a Daubechies 4 wave-let). After the DWT is applied, we computed features using the entire extracted time window for each signal (i.e., mapping each 60–200-min window to one set of features) by using a set of time-series measures. We selected 16 different candidate features (see Supplemental Table 1, Supplemental file 2, which list all features) across both complexity and temporal domains.

### Classification Framework

**Fig. 1:**
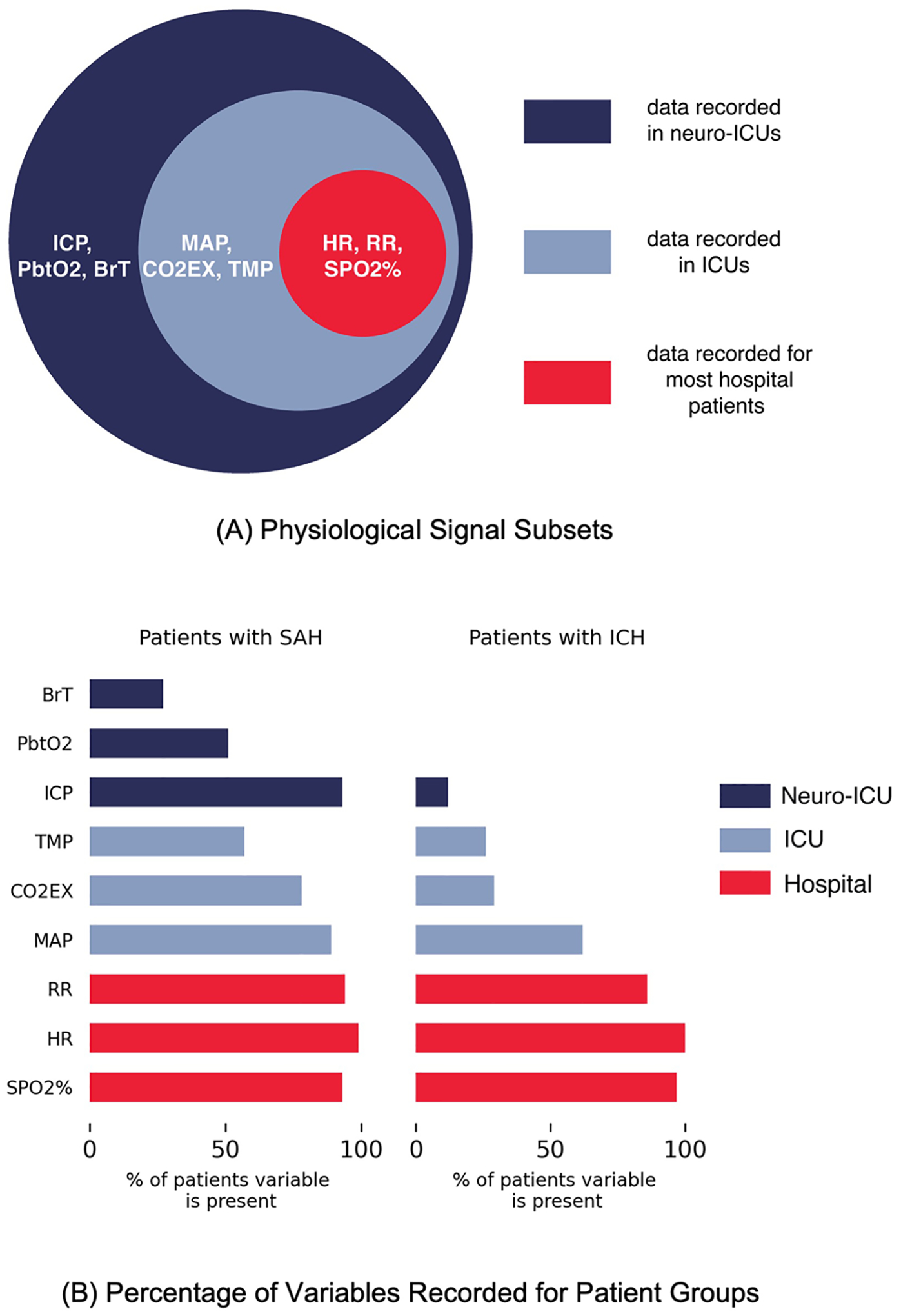
**A** Illustration of the physiological signals used in this study and related variable subsets; **B** Bar graph of the percentage of variables recorded for assessments in each patient group. Note that a signal is considered present when it is measured for at least 80% of the time window. SAH subarachnoid hemorrhage, ICH intracerebral hemorrhage, ICU intensive care unit, SPO2% blood oxygen level, HR heart rate, RR respiratory rate, MAP mean arterial pressure, CO2EX end-tidal carbon dioxide, TMP body temperature, ICP intracranial blood pressure, PbtO2 brain tissue oxygenation, and BrT brain temperature.

To perform classification, we used the XGBoost implementation of gradient boosted trees [27], which trains an ensemble of multiple weak learners (usually decision trees) sequentially to learn the errors of the previous models and then combines them to form a classification model. We chose this approach because an interpretable model is vital for this clinical application, and XGBoost is capable of learning from data where different features are present for different data samples. In our case, time windows may not have all physiological variables recorded (see Fig. 1b), and subsequently, only a subset of features will be present. Due to the differences in both data availability and disease severity, we perform classification on SAH and ICH patient groups separately.

### Model Training and Evaluation

**Fig. 2:**
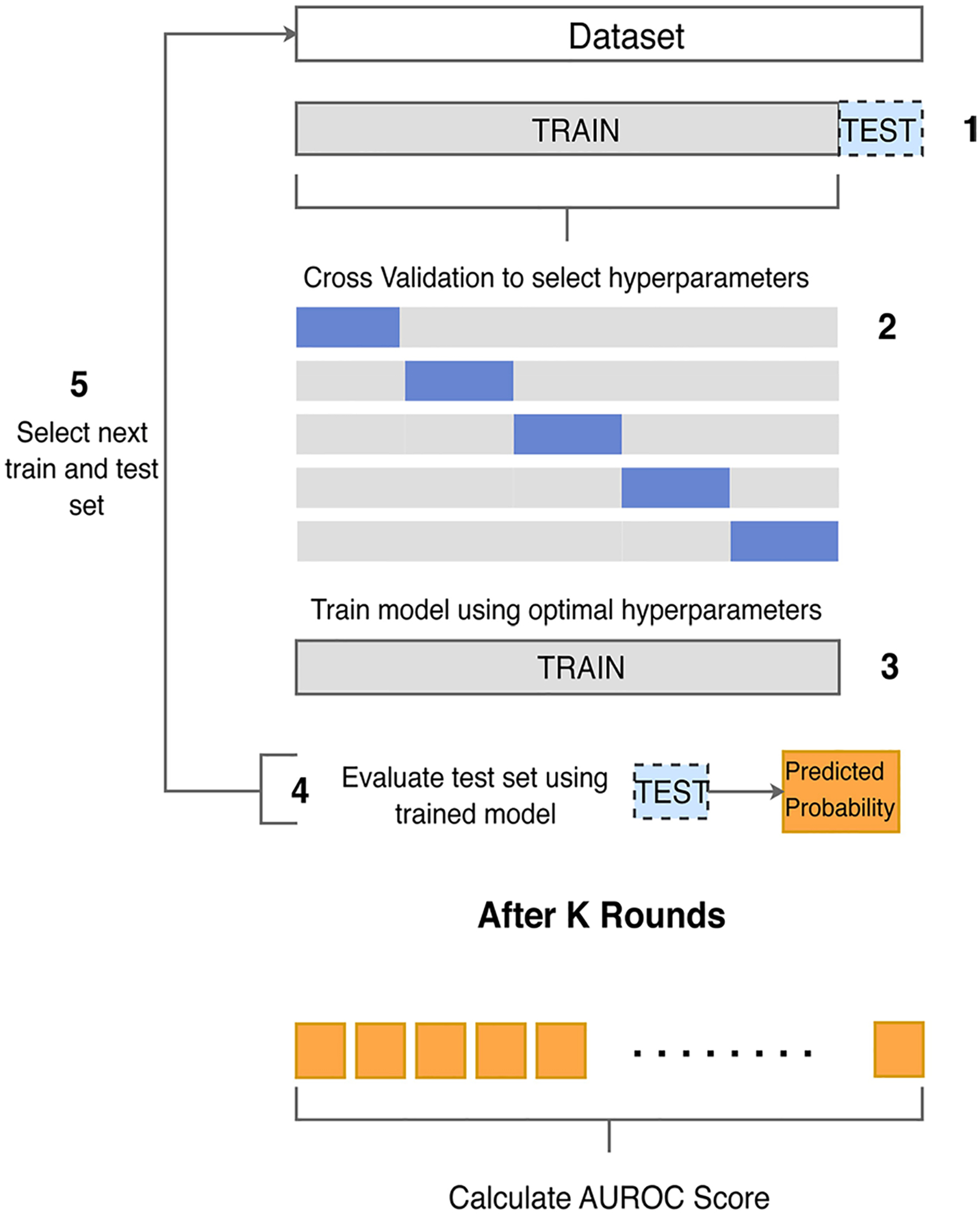
Classification framework using leave one patient out. (1) Leave one patient out (LOPO) as test set and train on the remaining data. (2) Perform 5-fold cross validation using train data to select hyperparameters including gamma, minimum child weight, maximum depth, and learning rate. (3) Use entire train data with learned hyperparameters to re-train the model. (4) Evaluate the held-out patient to obtain prediction probabilities. (5) Perform the same loop for K rounds. After K rounds, calculate the area under the receiver operating curve using K predicted probabilities. Note that the feature selection step is not shown in this figure.

We explore model performance under a nested cross-validation setting where we leave one patient out (LOPO) from each training round to evaluate our method without any training data from the test patient. During training, we performed hyperparameter tuning (see Supplemental file 1), feature selection (see Supplemental file 1), and rebalanced the data via a cost reweighting approach [28] by adding a positive weight (ratio of larger to smaller class sizes) to the samples in the smaller class, which penalizes its misclassification during training. Note that in each LOPO round, we train using only physiological signals measured for the held-out test patient because we want to evaluate using the same set of features. Figure 2 shows an overview of our classification framework for the LOPO scheme. We evaluated classification performance using multiple methods to capture various facets of performance, including: area under the precision recall curve (AUPRC), receiver operating characteristic (ROC) curves, area under the ROC curve (AUROC) with sensitivity and specificity at the operating point of the ROC curve (i.e., the point closest to the [0, 1] point) using Youden’s J statistic, confusion matrices (see Supplemental File 2, Supplemental Figs. 2 and 3), and accuracy (see Supplemental File 2, Supplemental Table 7). For each AUROC value, we report the 95% confidence interval (CI) using the DeLong approach [29]. Additionally, we provide information about model calibration (see Supplemental file 1 and Supplemental Fig. 1 in Supplemental file 2) using the LOW-ESS calibration curve, the Integrated Calibration Index [30], and the maximum absolute difference between the predicted and observed probability (*E*_*max*_) [31].

### Physiological Signal Feature Importance

To understand how each signal contributes to overall classification accuracy, we examined physiological signal feature importance using the top *k* important features with *k* = 5. Given our LOPO approach and the differences in signals that are measured for each patient, we define a measure of signal *relevance* to capture each signal’s impact on classification. This measure is modified from the approach introduced in Claassen et al. [24], focused on finding common causal relationships in which patients have different signals, measured to our case, in which different features are used in each training round. Signal relevance is defined as the ratio between the number of times a signal appears in the top *k* important features to the total number of times it was used in a training across all rounds. For example, if HR is measured in all patients but appeared in the top 5 features 30% of the time, it would have a relevance of 0.3. Note that we count each signal once regardless of how many times its features appeared in the top 5 important features for each round. For example, if the mean and skew of HR both appear in the top 5 important features for a training round, HR as a signal is only counted once. In general, we find that the signal relevance scores for most physiological signals vary across classification tasks, variable subsets, and patient populations. See Supplemental Table 8 (Supplemental File 2) for results.

### Experiments

We aimed to test whether continuously recorded physiological data can be used to classify levels of consciousness. As some variables are specific to the neuro-ICU, and assessing consciousness is important for all hospital patients, we conducted experiments with three subsets of variables as shown in Fig. 1a: variables collected for most hospital patients (hospital), variables collected mainly in ICUs (ICU), and variables specific to neuro-ICUs (neuro-ICU). The neuro-ICU subset contains both the ICU and hospital subsets, and similarly, the ICU subset also contains the hospital subset. The hospital subset includes HR, RR, and SPO2%. The ICU subset further includes variables that are recorded in a range of ICUs regardless of specialization (e.g., cardiac or surgical): MAP, TMP, and CO2EX. Finally, the neuro-ICU subset includes variables that are not common to other ICUs but that may be important for assessing neurological status. In prior work, we found that correlations among brain-related variables have longer time lags in patients with SAH with lower versus higher levels of consciousness [23]. Thus, we included the variables found to be important in that study: ICP, BrT, and PbtO2. Including the hospital and ICU subsets helps to understand what performance may be achievable without the specialized data available only in neuro-ICUs. Figure 1b shows the percentage of assessments from the patients with SAH and patients with ICH for whom each variable is recorded. Note that the ICH patient group does not have variables specific to the neuro-ICU due to the difference in severity and treatment plans for the two groups; hence, no experiments are performed using the neuro-ICU subset.

With these three subsets, we also have three classification tasks selected based on clinical relevance: (1) classification between VS/UWS and MCS−; (2) classification between (Coma, VS/UWS) and (MCS−, CF); and (3) classification between non-CF and CF. In task 1, we classified between VS/UWS and MCS− because patients who are MCS− have some evidence of being aware of themselves and their environment compared with patients who are VS/UWS. Additionally, this is a common task performed in the classification of consciousness literature [20, 21]. Task 2 is relevant to prognosis, as patients with MCS− or higher have a better chance of recovering consciousness. For task 3, the classification of CF tells us about patients who can process information and are aware of their environment through verbal or nonverbal behavior [15]. Although this problem can be naturally framed as a multiclass classification problem, this would require a larger set of training data. Thus, we use the clinically relevant binary tasks found in prior work.

## Results

**Fig. 3:**
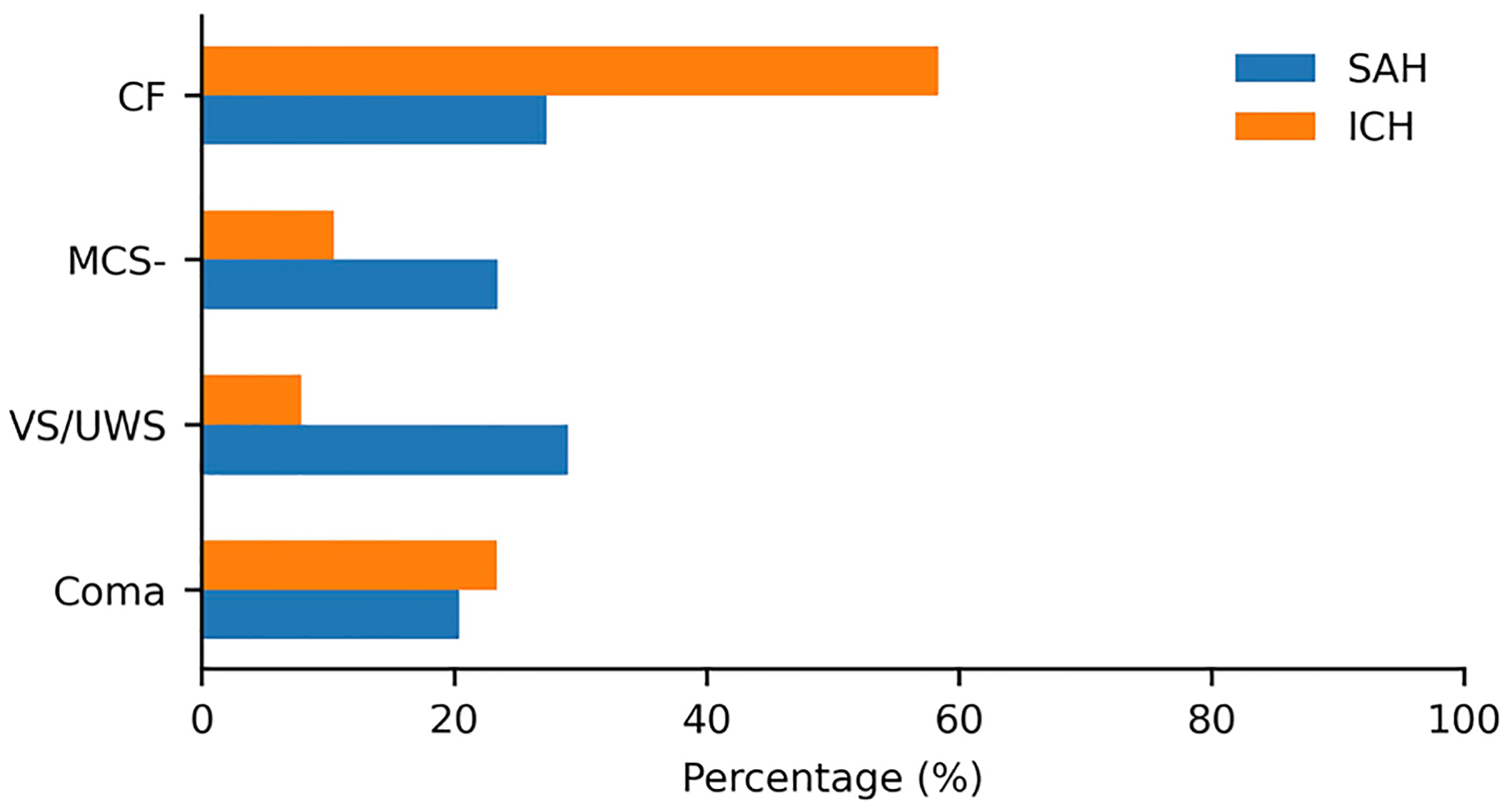
Bar graph showing the percentages of grouped behavioral scores for patients with SAH and ICH. SAH subarachnoid hemorrhage, ICH intracerebral hemorrhage, CF command following, MCS− minimally conscious state minus, and VS/UWS vegetative state/unresponsive wakefulness syndrome

**Fig. 4:**
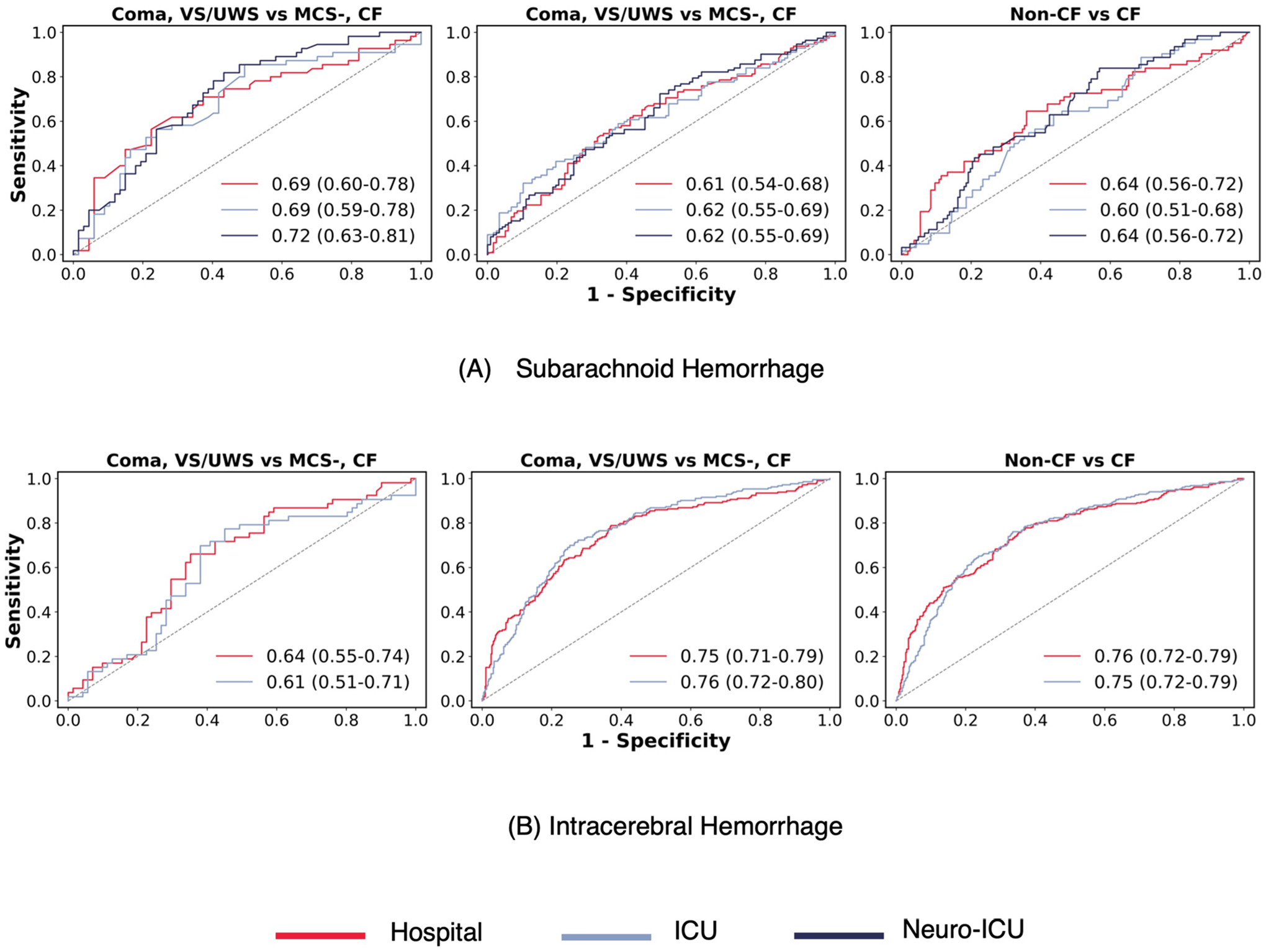
**A** ROC curves showing performance for each classification task and physiological data subset on the subarachnoid hemorrhage patient group for LOPO; **B** ROC curves showing performance for each classification task and physiological data subset on the intracerebral hemorrhage patient group for LOPO. VS/UWS vegetative state/unresponsive wakefulness syndrome, MCS− minimally conscious state minus, CF command following, ICU intensive care unit, Neuro-ICU neurological intensive care unit, and LOPO leave one patient out

We identified 61 patients with SAH and 180 patients with ICH that met the inclusion criteria of having both physiological signals and recorded behavioral assessments. In both patient groups, the number of assessments per patient is not equally distributed. In total, there are 1,815 assessments of consciousness (SAH: 267, ICH: 1,548) with an average of 7.53 assessments per patient (SAH: 4.38 ± 2.86, ICH: 8.6 ± 2.89). The minimum number of assessments per patient (SAH: 1, ICH: 2), maximum number of assessments (SAH: 14, ICH: 12), and inter-quartile range (SAH: 4.0, ICH: 5.0) also varied by group. After data processing and extracting time windows, 231 assessments from 61 patients with SAH and 698 assessments from 178 patients with ICH remained for further analysis. See Table 1 for characteristics of the studied population. There were more excluded assessments for patients with ICH due to the lack of recorded physiological signals after patients were discharged from the neuro-ICU. This breaks down into the following number of assessments for the predefined clinical categories: Coma (SAH: 47, ICH: 163); VS/UWS (SAH: 67, ICH: 55); MCS− (SAH: 54, ICH: 73); and CF (SAH: 63, ICH: 407). Figure 3 illustrates the distribution of behavioral scores, with a higher proportion of assessments of patients with ICH being in the CF category compared to the SAH group, where scores are more evenly distributed. See Supplemental Table 2 in Supplemental file 2 for the total number of samples in each of the classification tasks and across the variable subsets. For all experiments, we present classification performance in Fig. 4 with a ROC curve.

### VS/UWS Versus MCS−

In the SAH patient group, we achieved an AUROC of 0.72 (sensitivity: 82%, specificity: 57%; 95% CI 0.63–0.81) on the neuro-ICU subset. AUROC for this task using the ICU subset is 0.69 (sensitivity: 85%, specificity: 51%; 95% CI 0.59–0.78), while performance on the hospital subset is similar with an AUROC of 0.69 (sensitivity: 56%, specificity: 78%; 95% CI 0.60–0.78). Using AUPRC, we see improvements over the baseline of 0.45, with the best score of 0.72 on the neuro-ICU subset, 0.69 on the ICU subset, then 0.62 on the hospital subset. For patients with ICH, the best performance was obtained using the hospital subset with an AUROC of 0.64 (sensitivity: 66%, specificity: 65%; 95% CI 0.55–0.74), while the ICU subset had an AUROC of 0.61 (sensitivity: 77%, specificity: 55%; 95% CI 0.51–0.71). For the AUPRC, the best performance of 0.55 was on the hospital subset compared to 0.52 on the ICU subset, with both being improvements over the baseline of 0.43.

### (Coma, VS/UWS) Versus (MCS−, CF)

For this task, the AUROC increased marginally when we used more variables from the hospital subset with an AUROC of 0.61 (sensitivity: 56%, specificity: 65%; 95% CI 0.54–0.68) to 0.62 (sensitivity: 50%, specificity: 80%; 95% CI 0.55–0.69) on the ICU subset, and 0.62 (sensitivity: 72%, specificity: 50%; 95% CI 0.55–0.69) on the neuro-ICU subset for the SAH patient group. The AUPRC improved from the 0.49 baseline across all data subsets with scores of 0.59, 0.65, and 0.62 on the hospital, ICU, and neuro-ICU subsets. On the ICH patient group, the hospital subset had an AUROC of 0.75 (sensitivity: 79%, specificity: 63%; 95% CI 0.71–0.79) compared with 0.76 (sensitivity: 70%, specificity: 75%; 95% CI 0.72–0.80) on the ICU subset. The hospital subset had a higher AUPRC score of 0.60 compared with 0.57 on the ICU subset which are both improvements over the baseline score of 0.31.

### Non-CF Versus CF

In the SAH patient group, we observed the highest AUROCs of 0.64 (sensitivity: 65%, specificity: 64%; 95% CI 0.56–0.72) on the hospital subset and 0.64 (sensitivity: 84%, specificity: 43%; 95% CI 0.56–0.72) on the neuro-ICU subset. The ICU subset had an AUROC of 0.60 (sensitivity: 89%, specificity: 31%; 95% CI 0.51–0.68). The best AUPRC was on the hospital subset with a score of 0.40 compared to 0.34 on the ICU and 0.38 on the neuro-ICU subset. All scores achieved better performance than the baseline of 0.27. For patients with ICH, we found that results were similar to those of the prior task, with the hospital subset having an AUROC of 0.76 (sensitivity: 78%, specificity: 63%; 95% CI 0.72–0.79) compared to the AUROC of 0.75 (sensitivity: 76%, specificity: 66%; 95% CI 0.72–0.79) on the ICU subset. Both subsets improve over the baseline of 0.42 with AUPRC of 0.69 and 0.65 on the hospital and ICU subsets.

## Discussion

Methods that can automatically assess a patient’s level of consciousness could have a significant impact on patient care, reduce demands on a clinician’s time, and facilitate future research into why consciousness changes. Although previous research has examined the use of methods such as EEG, fEEG, and fMRI for assessing consciousness [32–34], there are major limitations to their use in providing the continuous measurements needed for effective brain monitoring. Our results across classification tasks showed that information from physiological signals may be associated with behavioral states of consciousness at the time of assessments. Further, we also examined how performance changes on the basis of which variables are used as we tested classification using variable subsets which included the minimal set of variables recorded for most hospital patients, variables measured in most ICUs, and an extended set recorded in neuro-ICUs. The crucial distinction between VS/UWS and MCS− was made by our approach, as evidenced by the AUROC of 0.72 on the SAH patient group, which compares favorably with prior studies that use technically challenging methods such as fEEG and fMRI (AUROC of 0.78 [21] and accuracy of at least 80% [35]). Although we achieved a lower AUROC value, our work is promising as we leverage continuously recorded physiological data from routinely used sensors, rather than requiring new sensors such as accelerometers [22]. Unlike other methods, our approach could potentially be used to provide more frequent and automated indicator for consciousness without needing to move patients, disrupt care, or invest in new technologies. Further, this allows for regular insights into when or if the level of consciousness is fluctuating which has been linked to worse outcomes (death, disability) at 3 months after SAH [14].

For all configurations, we achieved higher AUROC performance on the ICH patient group except on the first classification task (VS/UWS vs. MCS−) in which we attained a higher AUROC on the SAH patient group. This can be attributed to the greater likelihood that in the first task the SAH patient group has invasive monitoring which leads to more variables (and hence more information) being available for classification compared with the ICH patient group, whereas overall more assessments were available for the ICH group. On the second task (coma, VS/UWS vs. MCS−, CF) the highest AUROC on the ICH patient group is higher than that on ICH with our first task. This difference is likely due to the larger amount of training data available with the inclusion of CF labels (see Fig. 3) compared with the first task. This is even more apparent when considering our third task (non-CF vs. CF). In general, ICU variables are absent for many ICH assessments (see Fig. 1b and Supplemental Table 4 in Supplemental file 2). Thus, we did not expect to see a significant performance difference between the hospital and the ICU subset for all classification tasks. In the SAH patient group, there was an increase in AUROC from the hospital to the neuro-ICU subset across all classification tasks suggesting that the inclusion of additional variables helped capture more information that led to increases in predictive performance. On the AUPRC, we observe that for both the ICH and SAH patient groups, we consistently have scores higher than the baseline for each classification task.

### Consciousness and Physiological Signals

Our use of physiological signals expands previous research that focuses on patient state monitoring using the signals available in the ICU. This includes work in classifying levels of sedation [36, 37], predicting cardiac arrests [38], predicting the onset of sepsis [39, 40], and automating the measurement of pain intensity [41, 42] in ICUs. Like our methodology, these works either extract features from physiological signals or use them in their raw formats with a machine learning model to predict patient states that may assist clinicians in treating patients. Physiological signals have also been used for patient monitoring of consciousness in the ICU by associating changes in signals with changes in patient state. For example, a reduction in HR variability (HRV) is positively associated with a deepening coma state [43] and a higher complexity index value (HRV complexity score) in MCS patients compared with VS/UWS patients [44]. Additionally, other autonomic cardiac markers such as cardiac cycle have shown a significant phase shift in MCS patients compared with VS/UWS patients induced by global regularities in the local–global oddball paradigm [45]. Although our study does not focus on capturing changes in physiological signals associated with changes in consciousness levels (due to limited availability of ground truth data) these works show that such relationships may exist between the physiological variables we tested and different states of consciousness.

### Human Evaluation

Given our reliance on labeled assessments as ground truth, we discuss our results in context of how accurate clinicians are at classifying patients’ states of consciousness. This is important because if we can achieve comparable accuracy to clinicians, we can potentially reduce the time burden of performing assessments. The primary metrics for human assessment of consciousness focus on how often individuals agree, using interrater reliability and interdiagnostic agreement (measured using the kappa score *K*) on behavioral assessments such as the GCS [8], the FOUR [9], CF scale [10], and the CRS-R [11]. These studies use labeled data collected from a diverse set of clinicians (including neurologists, neuropsychologists, nurses, and other ICU staff) in an ICU setting. Overall, kappa scores ranged from 0.60–0.79 [11, 46–48] on the CRS-R, 0.68–0.83 [9, 49, 50] on the GCS, and 0.75–0.85 [9, 49, 50] on the FOUR scales. These scores indicate that clinicians have moderate to strong agreement in categorizing patients as having the same state of consciousness (e.g., MCS) or assigning the same behavioral assessment scores. Although a direct comparison between performance metrics (AUROC and kappa score) is not possible, prior work has derived a mathematical relationship between the kappa score and ROC curve^1^ [51]. Hence, our results on the various classification tasks, variable subsets, and patient groups show that we achieve equally good discriminative performance in classifying patients into states of consciousness.

### Limitations

Although our work serves as a first step toward investigating the use of physiological signals to correlate behavioral states of consciousness, there are some limitations. First, although other works rely on well-studied features like EEG biomarkers [20, 21], our approach examined a range of time-series measures for classification. We found that across both classification tasks and patient groups, different features were selected for classification (see Supplemental file 1). Hence, it remains to be determined what features may be broadly informative across tasks and ICU types. Second, we faced a high degree of missing physiological signals for both the SAH and ICH patient groups when performing classification. Missingness varied substantially by signal, with some variables having high rates of missingness, TMP (SAH: 43%, ICH: 74%), others differing significantly by patient group, ICP (SAH: 7%, ICH: 88%), and finally, some variables having very low missingness (SPO2% SAH: 7%, ICH: 3%; HR SAH: 1%, ICH: 0%). We partly accounted for this by imputing missing values within signals (before extracting time windows) and using a model that can handle missing features with the LOPO cross-validation approach; however, this limits our ability to learn one general predictive model. Future work could examine other methods of learning shared information from patient data to better learn a general model. Further, although our imputation approach, Fourier Lagged *k*-nearest neighbors, has been previously validated on this data, it is a single imputation method and thus there may be variability in our results due to the imputation step. Third, we analyzed a limited number of patients and assessments. We accounted for this by using the LOPO cross-validation approach, which is best suited for cases with a limited number of samples and reported the AUROC (with confidence intervals), AUPRC, and model calibration (see Supplemental file 1 and Supplemental Fig. 1 in Supplemental file 2 for results) to further show the reliability of our results on both patient groups. In the future, we plan to collect more data from other ICUs to increase the size of our datasets and further test generalizability. Fourth, we used signals from the 60 to 200 min prior to each assessment across all tasks and patient groups, with assessments occurring daily during the morning. Because current practice is to conduct assessments during morning rounds, we were not able to compare performance to assessments from other times of the day. However, future work is needed to determine whether there are differences related to circadian rhythms or other time of day effects. Similarly, it remains to determine what the optimal window size is for each task, but this may be determined experimentally in the future with larger data sets. Lastly, the use of physiological signals enables continuous classification of consciousness, but we are limited by the availability of ground truth labels. Although our current approach can be used to perform classification at any time point, we only know if the classified states of consciousness are correct at the time of behavioral assessments. In our future work, we aim to expand the set of ground truth labels with more frequent assessment to capture fluctuations in consciousness. As assessments are labor intensive and involve the removal of sedation, it is infeasible to collect them at a high frequency, so future work may also involve using simulation for robust evaluation of classification and capturing fluctuations in consciousness.

## Conclusions

In this study, we demonstrated that physiological signals may be associated with behavioral states of consciousness. Although our work is preliminary, given our study limitations, it allows for further extensions to address these challenges and other works that similarly examine whether widely available physiological signals could serve as measurements that correlate with assessments of consciousness. These measurements can form the basis of a clinical alarm system that alerts clinicians to possible changes in neurological status with high sensitivity and specificity, triggering a bedside assessment and furthering diagnostic studies for therapeutic interventions. We aim to extend this work beyond the neuro-ICU to other ICUs where patients have impaired consciousness.

## Supplementary Material

Supplemental file 1

Supplemental file 2

## Figures and Tables

**Table 1:** Demographic and clinical characteristics for patients studied

Characteristics	SAH (n = 61)	ICH (n = 178)
Demographics		
Age	54 (45–63)	68 (56–77)
Female sex	44 (72)	73 (41)
Clinical/radiographic		
Glasgow coma scale	7 (4–9)	12 (7–15)
Acute physiology and chronic health evaluation II score	21 (18–26)	15 (9–20)
ICH score	NA^a^	2 (1–3)
Hunt-Hess scale on admission	4 (4–5)	NA
Delayed cerebral ischemia	21 (34)	–
Intraventricular hemorrhage sum score	4.0 (1.0–7.0)	NA
Global cerebral edema	44 (72)	–
Fischer score	3.00 (3.00–3.00)	NA
ICH location^b^		
Deep (thalamus/basal ganglia)	–	83 (53)
Lobar	–	50 (32)
Infratentorial	–	24 (15)
External ventricular drain placement	52 (85)	37 (21)
Clipping	52 (85)	0 (0)
Coiling	12 (20)	0 (0)
Invasive intracranial monitoring	61	22

Data are shown as count (%) or median (25th percentile–75th percentile) ICH, intracerebral hemorrhage, SAH, subarachnoid hemorrhage

a NA means not available

b ICH location is not present in patients with SAH and absent in 21 patients with ICH

## Footnotes

1 This relationship is defined as:

$$K=\frac{TP-FP-P^{\prime }\left(1-2N\right)}{P-P^{\prime }\left(1-2N\right)}$$

which outputs the kappa score (*K*) for a specified true positive (*TP*) and false positive, (*FP*) value on the ROC curve (with the simplified representation: *TP* = *f* (*FP*)). Here *P* is the number of positive labels, *N* is the number of negative labels, *P*′ is the probability of being predicted as positive, and *f* is the function that outputs true positive values for each false positive.
