# Supplementary material for: Classification of Level of Consciousness in a Neurological ICU Using Physiological Data
Source: Neurocrit Care. Author manuscript; Available in PMC 2023 Feb 25. (PMC9935697; doi:10.1007/s12028-022-01586-0)
Supplement: Supplemental file 1 [file NIHMS1854701-supplement-Supplemental_file_1.docx]

SUPPLEMENTAL MATERIAL

**Imputation**

To impute missing data, we used the Fourier lagged k nearest neighbor ^1^ approach which is a combination of the Fourier transform and lagged k nearest neighbor imputation methods. We impute using this method as it handles missing data in the case where variables are both missing at random (MAR), and not missing at random (NMAR); which are missing mechanisms inherent in our dataset. The parameters of the method include the number of nearest neighbors (k = 5), the maximum time lag (L = 60 minutes), and the number of lags for each variable pair (p = 3).

**Feature Extraction**

To remove high frequency noise (after filtering for outliers), we used a level 2 discrete wavelet transform with the Daubechies 4 wavelet. The resultant approximation coefficient is then used to compute features. We compute 16 features over the approximation coefficients listed in Supplemental Table 1. Note that *stability* and *non-linearity* were computed using the tsfeatures ^2^ time series package. All other features are computed using either NumPy ^3^, SciPy ^4^ or nolds ^5^ which all have module for computing features of time series data.

**Feature Selection**

To select the most predictive features for classification, we used the sequential forward feature selection algorithm with the AUROC as the scoring metric for evaluating performance of the feature combinations. Feature selection was performed independently for each classification task, data subset and for the SAH and ICH patient groups, which means that potentially different features are selected and deemed as the important in each configuration. We examine this by looking at the physiological signals and time series measures that are deemed as important or relevant in each experiment. The result for physiological signals relevance is presented in Supplemental Table 8 (supplemental file 2), so here we highlight the time series measures that were consistently found in the important features used for classification.

VS/UWS vs MCS-

On the SAH patient group, the time series measures include the hospital subset: Shannon entropy, kurtosis, linearity, and median difference; ICU subset: kurtosis, skew, mean absolute deviation, and interquartile range (IQR); and Neuro-ICU subset: range, absolute energy, IQR, Higuchi dimension. For the ICH patient group, these include hospital subset: standard deviation (SD), linearity, stability, and Higuchi dimension; and ICU subset: Hurst coefficient, Higuchi dimension, and coefficient of variation (CV).

(Coma, VS/UWS) vs (MCS-, CF)

For the SAH patient group, the time series measures on each subsets include hospital subset: IQR, SD, range, and entropy; ICU subset: stability, Shannon entropy, absolute energy, range, and absolute energy; and Neuro-ICU subset: range, SD, Hurst coefficient and absolute energy. For the ICH patient group, these include hospital subset: Shannon entropy, range, IQR, and mean absolute deviation; and ICU subset: IQR, root mean square (RMS), and absolute energy.

Non-Command Following vs Command Following

For the SAH patient group, time series measures selected include the hospital subset: CV, mean absolute deviation, and mean absolute deviation; ICU subset: Shannon entropy, CV, and Higuchi dimension; and Neuro-ICU subset: skew, mean absolute deviation, and range. For the ICH patient group, the hospital and ICU subsets had Shannon entropy, and SD in common, except for RMS and kurtosis in the hospital subset and absolute energy in the ICU subset.

**Hyperparameter Tuning**

For our classification framework, in each round, we hold out either a single assessment of consciousness or all the assessments of consciousness for a given patient. The held-out portion is the test data, and the rest are considered the training dataset. We performed hyperparameter tuning to learn the best parameters for our model on the training dataset in each round. While XGBoost has several hyperparameters that can be tuned, we selected hyperparameters that are closely related to controlling for overfitting. These include the maximum depth of trees, minimum child weight, gamma, and the learning rate. The maximum depth of trees is the maximum depth (from the root node) of the boosted trees created by the model. The larger it is, the more our model is likely to overfit on the training dataset. Minimum child weight is the minimum sum of weights required for further partitioning of leaf nodes. If the sum of weights is less than the set minimum child weight, no further partitioning of the node is done. Gamma is the threshold value that needs to be met before further partitioning takes place. If the difference in the new loss and prior loss from the cost function is not greater than gamma, no further partitioning takes place. The learning rate is used to scale the weights of the features. Smaller values of eta reduce feature weights and make learning more conservative. We optimized for the best AUROC by using 5-fold cross-validation (on the training dataset after performing feature selection) to select the best combination of hyperparameters, and early stopping to restrict the number of boosted trees. We first performed a grid search over values for the maximum depth of trees, minimum child weight, and gamma. We searched over hyperparameter values from 2 to 7 for both the maximum depth of trees and minimum child weight, values from 1 to 4 for gamma, and value of 0.1 and 0.01 for the learning rate. The number of boosted trees used is the number of boost rounds needed to achieve the optimum AUROC from the optimization step.

**Model Calibration**

Overall, our results on model calibration show that our model achieves calibration curves close to the perfect calibration line based on the Integrated Calibration Index (ICI) and E-max metrics except in the cases where there is either few samples (classification between VS/UWS vs MCS- for SAH and ICH patient groups) or when there is a major class imbalance (Non-CF vs CF for the SAH patient group). Future work will focus on performing classification with larger number of patients to address both low sample size and data imbalance to improve calibration metrics. See Supplemental Figures 1 in Supplemental file 2 for the LOWESS calibration curves and associated ICI and E-max values.

REFERENCES

1. Rahman SA, Huang Y, Claassen J, Heintzman N, Kleinberg S. Combining Fourier and lagged k -nearest neighbor imputation for biomedical time series data. J Biomed Inform 2015;58:198–207.

2. Rob Hyndman and Yanfei Kang and Pablo Montero-Manso and Thiyanga Talagala and Earo Wang and Yangzhuoran Yang and Mitchell O’Hara-Wild. tsfeatures: Time Series Feature Extraction [Internet]. 2022. Available from: https://pkg.robjhyndman.com/tsfeatures/

3. Harris CR, Millman KJ, van der Walt SJ, et al. Array programming with NumPy. Nature 2020;585(7825):357–62.

4. Virtanen P, Gommers R, Oliphant TE, et al. SciPy 1.0: fundamental algorithms for scientific computing in Python. Nat Methods 2020;17(3):261–72.

5. Schölzel, Christopher. Nonlinear measures for dynamical systems [Internet]. Zenodo; 2019 [cited 2022 Apr 18]. Available from: https://zenodo.org/record/3814723

6. Claassen J, Rahman SA, Huang Y, et al. Causal Structure of Brain Physiology after Brain Injury from Subarachnoid Hemorrhage. PLOS ONE 2016;11(4):e0149878.
