# Supplementary material for: Classification of Level of Consciousness in a Neurological ICU Using Physiological Data
Source: Neurocrit Care. Author manuscript; Available in PMC 2023 Feb 25. (PMC9935697; doi:10.1007/s12028-022-01586-0)
Supplement: Supplemental file 2 [file NIHMS1854701-supplement-Supplemental_file_2.docx]

Supplemental File for the Classification of Level of Consciousness in a Neurological ICU using Physiological Data

|  | Description | Page(s) |
| --- | --- | --- |
| Table S1 | Time Series Features we consider for Classification | 2 |
| Table S2 | Number of Samples in each Variable Subset for each Classification Task across Patient Groups. | 3 |
| Table S3 | Physiological Signals, Filter Ranges for Outliers, and their Respective Monitoring Devices. | 4 |
| Table S4 | Percentage of Variables Missing after Time Windows Extraction. | 5 |
| Table S5 | AUROC for both Patient Groups across Variable Subsets using the LOPO Framework on all Classification Tasks | 6 |
| Table S6 | AUPRC for both Patient Groups across Variable Subsets using the LOPO Framework on all Classification Tasks | 7 |
| Table S7 | Accuracy for both Patient Groups across Variable Subsets using the LOPO Framework on all Classification Tasks | 8 |
| Fig S1 | Model Calibration Curves for the LOPO Framework on all Classification Tasks and Patient Groups | 9 |
| Table S8 | Physiological Signal Relevance for all Signals across both Patient Groups, Variable Subsets and Classification Tasks on the LOPO framework*.* | 10 |
| Fig S2 | Confusion Matrix showing Performance for each Classification Task and Physiological Variable Subsets on the SAH Patient Group for the LOPO framework. | 11,12 |
| Fig S3 | Confusion Matrix showing Performance for each Classification Task and Physiological Variable Subsets on the ICH Patient Group for the LOPO framework. | 13,14 |

Supplemental Table 1: Time Series Features we consider for Classification

| Domain | Features |
| --- | --- |
| Temporal | Mean, Absolute energy, Kurtosis, Skew, Root mean square, Coefficient of variation, Range, interquartile range, Standard deviation, Mean absolute deviation, Median difference, Stability, Nonlinearity |
| Complexity | Hurst Coefficient, Higuchi Dimension, Shannon Entropy |

Supplemental Table 2: Number of samples in each variable subset for each classification task across patient groups.

|  | Patient Groups and Variable Subsets | | | | |
| --- | --- | --- | --- | --- | --- |
| Classification Tasks | SAH | | | ICH | |
|  | Hospital | ICU | Neuro-ICU | Hospital | ICU |
| UWS/VS vs MCS- | 121 | 121 | 121 | 128 | 128 |
| Coma, UWS/VS vs MCS-, CF | 231 | 231 | 231 | 698 | 697 |
| Non-CF vs CF | 231 | 231 | 231 | 698 | 697 |

SAH = subarachnoid hemorrhage, ICH = intracerebral hemorrhage, CF = command following, MCS- = minimally conscious state minus, and VS/UWS = vegetative state/unresponsive wakefulness syndrome, ICU = intensive care unit.

| Physiological Signals  [unit] | Outlier Filter Range | Monitoring Devices, measuring technique |
| --- | --- | --- |
| SPO2, blood oxygenation | X <= 100 | Covidien Nellcore |
| HR, heart rate [beats per minute] | 0 < X < 350 | General Electric Solar 8000i monitors |
| RR, respiratory rate [breath/min] | 0 < X < 50 | 840-Puritan Bennett^TM^, Covidien |
| MAP, mean arterial pressure [mmHg] | 20 < X < 200 | General Electric Solar 8000i monitors |
| CO2EX, end-tidal carbon dioxide [mmHg] | 0 < X < 200 | Infrared capnometer, Respironics^TM^, Philips |
| TMP, body temperature [degrees Celsius] | 20 < X < 45 | Bladder temperature probe, Bardex^TM^, Bard Medical |
| ICP, intracranial pressure [mmHg] | 0 <= X < 200 | Integra Neurosciences^TM^ |
| PbtO2, brain tissue oxygenation [mmHg] | Exclude values above 95^th^ percentile for each subject | LICOX^TM^, Integra Neurosciences |
| BrT, brain temperature [degrees Celsius] | 30 < X < 45 | Bowman Perfusion Monitor^TM^, Hemedex |

Supplemental Table 3: Physiological signals, filter ranges for outliers, and monitoring devices used. Note that outlier filtering is applied independently for each patient.

Supplemental Table 4: Percentage of variables missing after time window extraction.

| Physiological Signals | Percentage of extracted windows where variable is absent (%)^1^ | |
| --- | --- | --- |
|  | SAH  (n = 229 assessments) | ICH  (n = 681 assessments) |
| SPO2, blood oxygenation | 7 | 3 |
| HR, heart rate | 1 | 0 |
| RR, respiratory rate | 6 | 14 |
| MAP, mean arterial pressure | 11 | 38 |
| CO2EX, end-tidal carbon dioxide | 22 | 71 |
| TMP, body temperature | 43 | 74 |
| ICP, intracranial pressure | 7 | 88 |
| PbtO2, brain tissue oxygenation | 49 | - |
| BrT, brain temperature | 73 | - |

^1^ Variables are considered absent if they are not recorded for at least 80% of the time window

SAH = subarachnoid hemorrhage, ICH = intracerebral hemorrhage

Supplemental Table 5: AUROC for both patient groups across variable subsets using the LOPO framework on all classification tasks

| Classification Tasks | Patient Groups | AUROC (95% CI) | | |
| --- | --- | --- | --- | --- |
|  |  | Hospital Data | ICU Data | Neuro-ICU data |
| Vegetative/Unresponsive Wakefulness  Vs  Minimally Conscious state-minus | SAH | 0.69 (0.60-0.78) | 0.69 (0.59-0.78) | 0.72 (0.63-0.81) |
|  | ICH | 0.64 (0.55-0.74) | 0.61 (0.51-0.71) | - |
| Coma, Vegetative/ Unresponsive Wakefulness  Vs  Minimally Conscious state-minus, Command Following | SAH | 0.61 (0.54-0.68) | 0.62 (0.55-0.69) | 0.62 (0.55-0.69) |
|  | ICH | 0.75 (0.71-0.79) | 0.76 (0.72-0.80) | - |
| Non-command Following  Vs  Command Following | SAH | 0.64 (0.55-0.73) | 0.60 (0.52-0.68) | 0.64 (0.56-0.72) |
|  | ICH | 0.76 (0.72-0.79) | 0.75 (0.72-0.79) | - |

SAH = subarachnoid hemorrhage, ICH = intracerebral hemorrhage, ICU = intensive care unit, Neuro-ICU = Neurological intensive care unit, AUROC = area under the receiver operating characteristic curve, LOPO = leave one patient out, and CI = confidence interval.

Supplemental Table 6: AUPRC for both patient groups across variable subsets using the LOPO framework on all classification tasks. Baseline for each task is the ratio of positive predictions in parenthesis under patient group.

| Classification Tasks | Patient Groups  (Baseline) | AUPRC | | |
| --- | --- | --- | --- | --- |
|  |  | Hospital Data | ICU Data | Neuro-ICU data |
| Vegetative/ Unresponsive Wakefulness  Vs  Minimally Conscious state-minus | SAH  (0.45) | 0.62 | 0.69 | 0.72 |
|  | ICH  (0.43) | 0.55 | 0.52 | - |
| Coma, Vegetative/ Unresponsive Wakefulness  Vs  Minimally Conscious state-minus, Command Following | SAH  (0.49) | 0.59 | 0.65 | 0.62 |
|  | ICH  (0.31) | 0.60 | 0.57 | - |
| Non-command Following  Vs  Command Following | SAH  (0.27) | 0.4 | 0.34 | 0.38 |
|  | ICH  (0.42) | 0.69 | 0.65 | - |

SAH = subarachnoid hemorrhage, ICH = intracerebral hemorrhage, ICU = intensive care unit, Neuro-ICU = neurological intensive care unit, AUPRC = area under the precision recall curve, and LOPO = leave one patient out.

Supplemental Table 7: Accuracy for both patient groups across variable subsets using the LOPO framework on all classification tasks. For all cases, the threshold for classification is 0.5.

| Classification Tasks | Patient Groups | Accuracy | | |
| --- | --- | --- | --- | --- |
|  |  | Hospital Data | ICU Data | Neuro-ICU data |
| Vegetative/ Unresponsive Wakefulness  Vs  Minimally Conscious state-minus | SAH | 0.68 | 0.65 | 0.67 |
|  | ICH | 0.64 | 0.60 | - |
| Coma, Vegetative/ Unresponsive Wakefulness  Vs  Minimally Conscious state-minus, Command Following | SAH | 0.60 | 0.60 | 0.58 |
|  | ICH | 0.72 | 0.72 | - |
| Non-command Following  Vs  Command Following | SAH | 0.66 | 0.62 | 0.66 |
|  | ICH | 0.70 | 0.69 | - |

SAH = subarachnoid hemorrhage, ICH = intracerebral hemorrhage, ICU = intensive care unit, Neuro-ICU = neurological intensive care unit, AUPRC = area under the precision recall curve, and LOPO = leave one patient out.


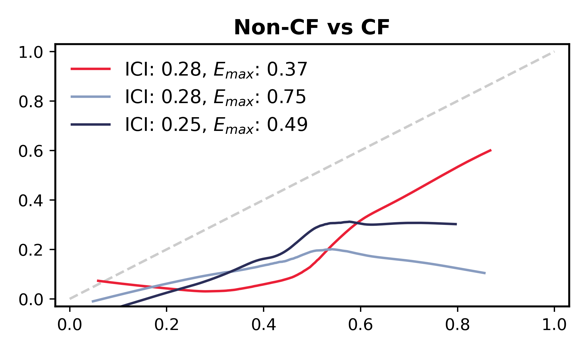

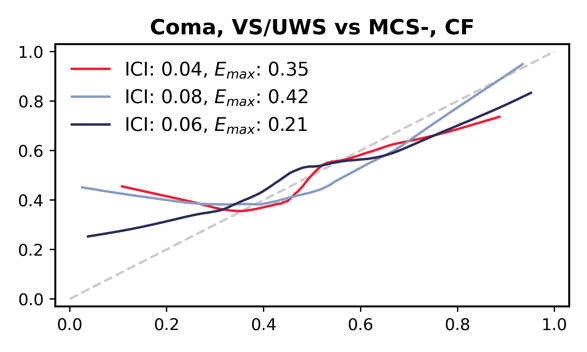

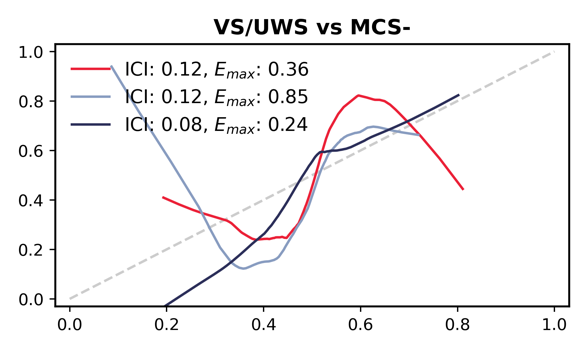


1. Subarachnoid Hemorrhage

**Observed Probability**


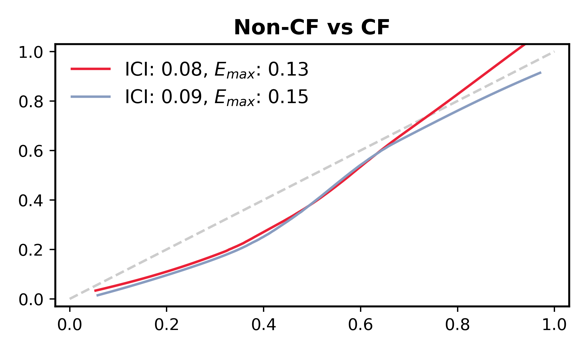

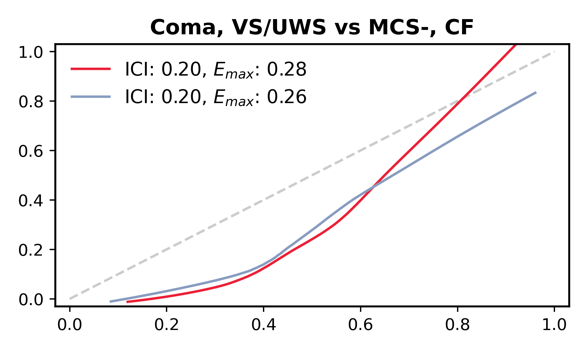

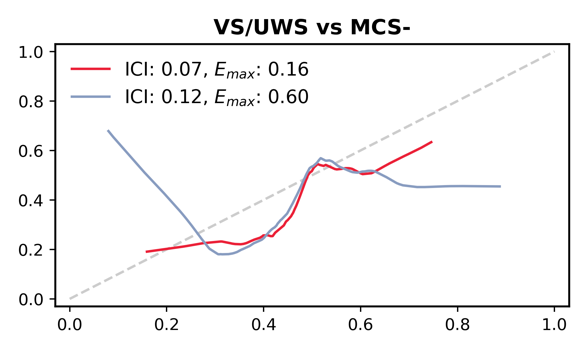


(B) Intracerebral Hemorrhage

**Predicted Probability**

Hospital

ICU

Neuro-ICU

**Figure S1:** Model Calibration Curves with ICI and $E_{max}$ for each classification task and physiological data subsets across both the (A) subarachnoid hemorrhage and (B) intracerebral hemorrhage patient groups on the LOPO framework. VS/UWS vegetative state/unresponsive wakefulness syndrome, MCS- minimally conscious state minus, CF command following, ICU intensive care unit, and Neuro-ICU neurological intensive care unit, SAH subarachnoid hemorrhage, ICH intracerebral hemorrhage, LOPO leave one patient out, ICI integrated calibration index, $E_{max}$ maximum difference between the predicted and observed probability.

Supplemental Table 8: Physiological signal relevance for all signals across patient groups, variable subsets, and classification tasks on the LOPO framework.

|  | | | Signal Relevance for Physiological Variables | | | | | | | | |
| --- | --- | --- | --- | --- | --- | --- | --- | --- | --- | --- | --- |
| VS/UWS vs MCS- | Patient Groups | Variable Subsets | HR | RR | SPO2% | CO2  EX | MAP | TMP | BrT | ICP | PbtO2 |
|  | SAH | Hospital | 0.9 | 0.92 | 0.96 | - | - | - | - | - | - |
|  |  | ICU | 0.12 | 0.88 | 0.96 | 0.86 | 0.96 | 0.03 | - | - | - |
|  |  | Neuro-ICU | 0.14 | 0.47 | 0.98 | 0.37 | 0.78 | 0.0 | 0.21 | 0.94 | 0.0 |
|  | ICH | Hospital | 0.98 | 0.71 | 0.98 | - | - | - | - | - | - |
|  |  | ICU | 1.0 | 0.56 | 1.0 | 0.0 | 0.15 | 0.0 | - | - | - |
|  | | | | | | | | | | | |
| Coma, VS/UWS vs  MCS-, CF | SAH | Hospital | 0.98 | 1.0 | 1.0 | - | - | - | - | - | - |
|  |  | ICU | 0.95 | 0.82 | 0.55 | 0.50 | 1.0 | 0.44 | - | - | - |
|  |  | Neuro-ICU | 1.0 | 0.27 | 0.53 | 0.60 | 1.0 | 0.30 | 0.04 | 0.07 | 0.0 |
|  | ICH | Hospital | 0.98 | 1.0 | 1.0 | - | - | - | - | - | - |
|  |  | ICU | 0.82 | 1.0 | 0.96 | 1.0 | 0.23 | 0.40 | - | - | - |
|  | | | | | | | | | | | |
| Non-CF vs CF | SAH | Hospital | 0.98 | 0.97 | 0.95 | - | - | - | - | - | - |
|  |  | ICU | 0.58 | 0.97 | 0.95 | 0.31 | 0.54 | 0.56 | - | - | - |
|  |  | Neuro-ICU | 0.88 | 0.43 | 0.81 | 0.92 | 0.43 | 0.72 | 0.0 | 0.14 | 0.0 |
|  | ICH | Hospital | 0.79 | 1.0 | 1.0 | - | - | - | - | - | - |
|  |  | ICU | 0.31 | 1.0 | 0.99 | 1.0 | 0.61 | 0.19 | - | - | - |

SAH = subarachnoid hemorrhage, ICH = intracerebral hemorrhage, ICU = intensive care unit, Neuro-ICU = neurological intensive care unit, LOPO = leave one patient out, VS = vegetative state, UWS = unresponsive wakefulness state, MCS- = minimally conscious state minus, CF = command following, SPO2% = blood oxygen level, HR = heart rate, RR = respiratory rate, MAP = mean arterial pressure, CO2EX = end-tidal carbon dioxide, TMP = body temperature, ICP = intracranial blood pressure, PbtO2 = brain tissue oxygenation, and BrT = brain temperature

**True Label**


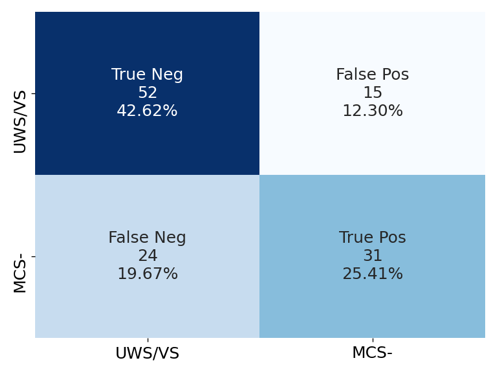

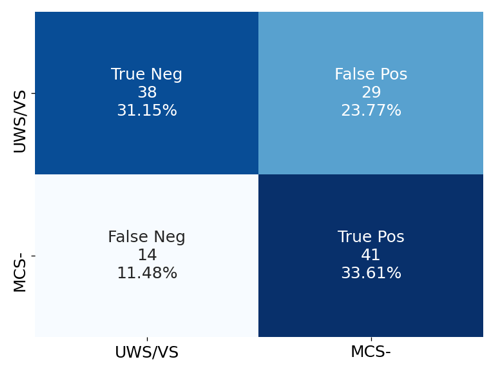

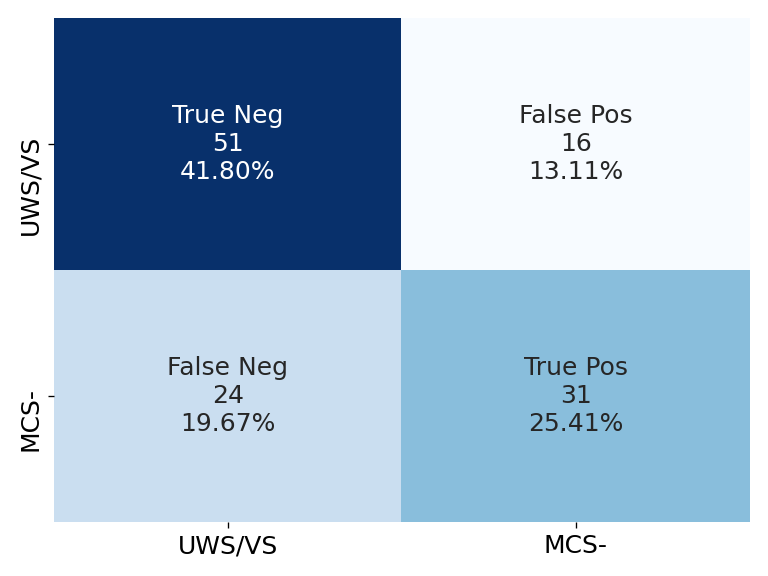


1. SAH, UWS/VS vs MCS-


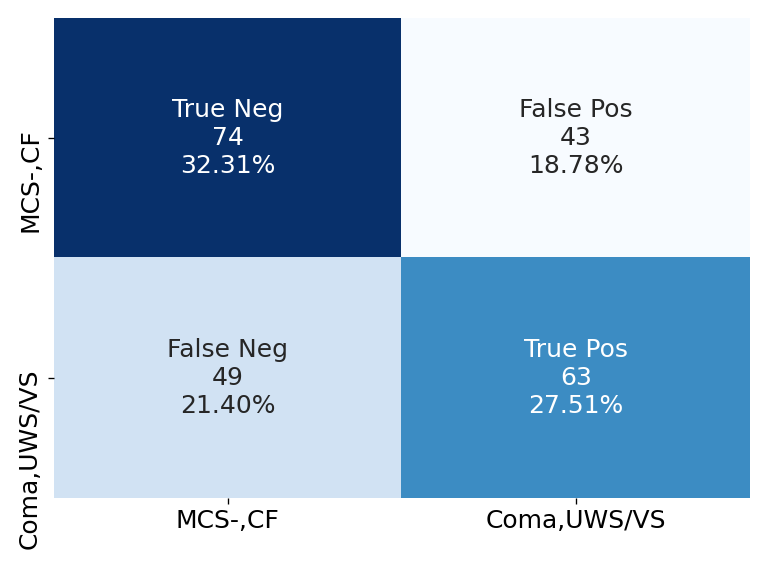

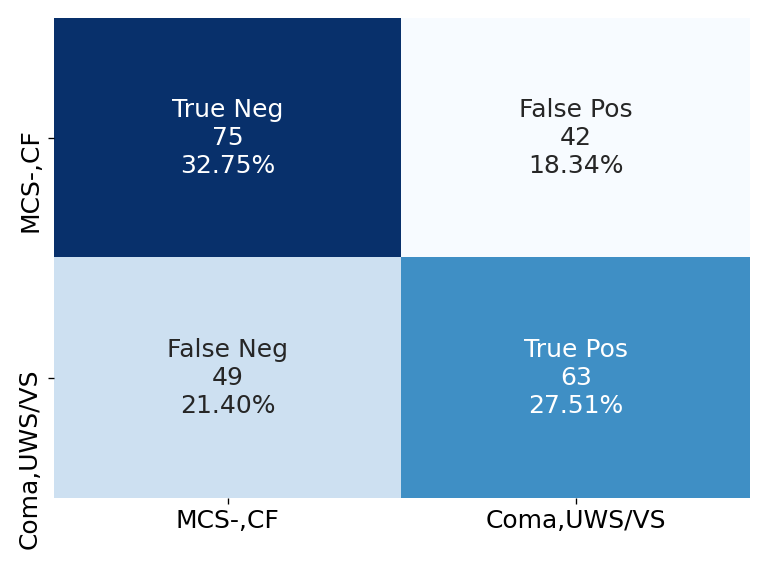

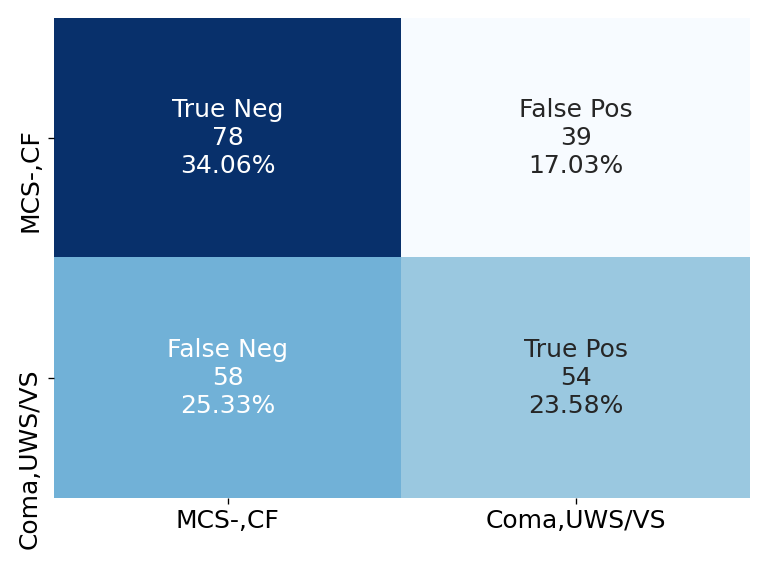


1. SAH, Coma, UWS/VS vs MCS-, CF


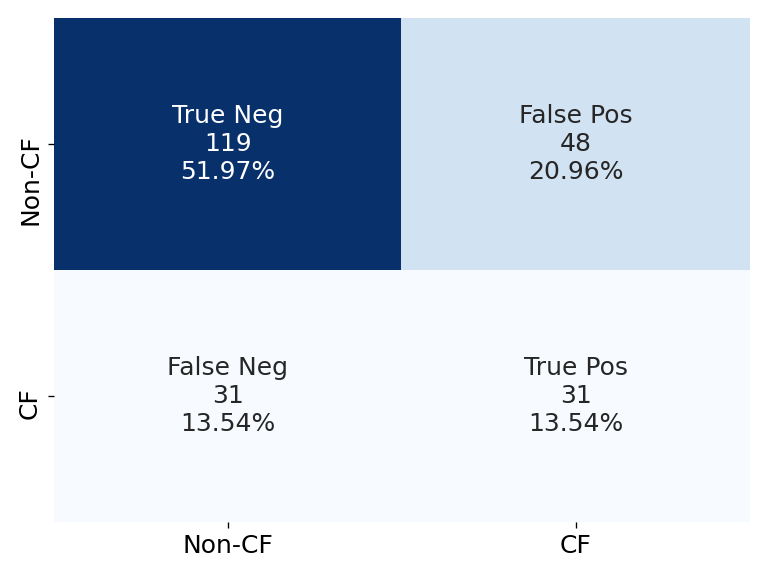

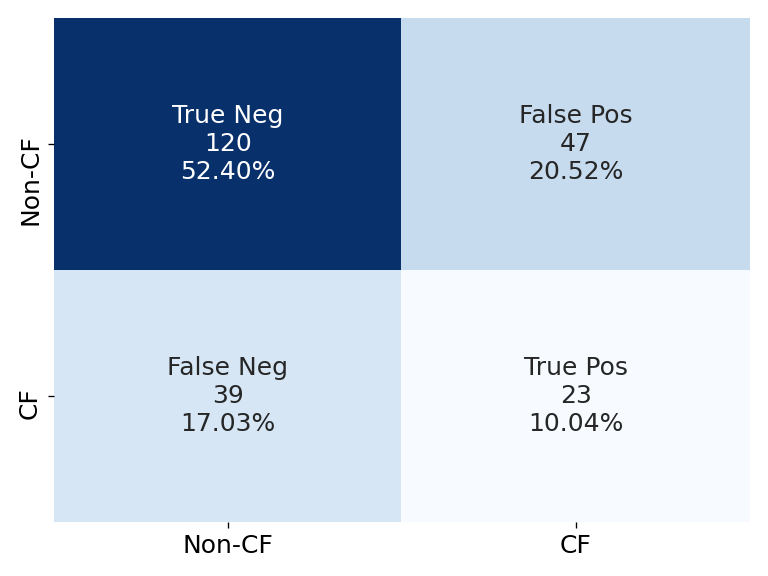

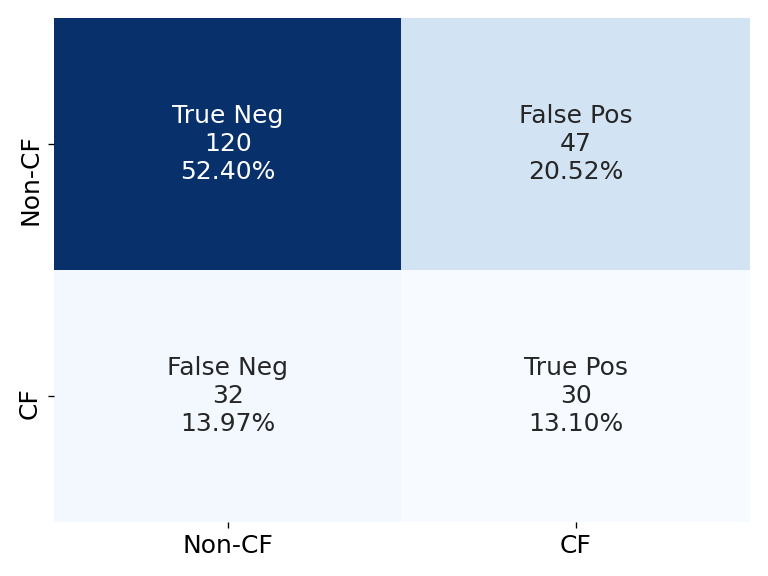


1. SAH, Non-CF vs CF

**Predicted Label**

**Hospital**

**ICU**

**Neuro-ICU**

**Figure S2:** Confusion matrix showing performance for each classification task (from top to bottom) and physiological variable subsets (from left to right) on the SAH patient group for the LOPO framework. VS/UWS = vegetative state/unresponsive wakefulness syndrome, MCS- = minimally conscious state minus, CF = command following, and LOPO = leave one patient out, SAH = subarachnoid hemorrhage, ICU = intensive care unit.

**Hospital**

**ICU**


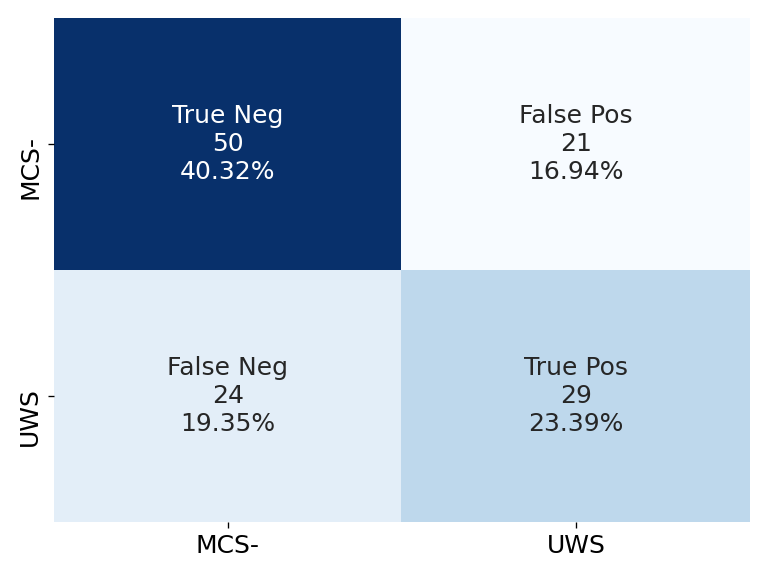

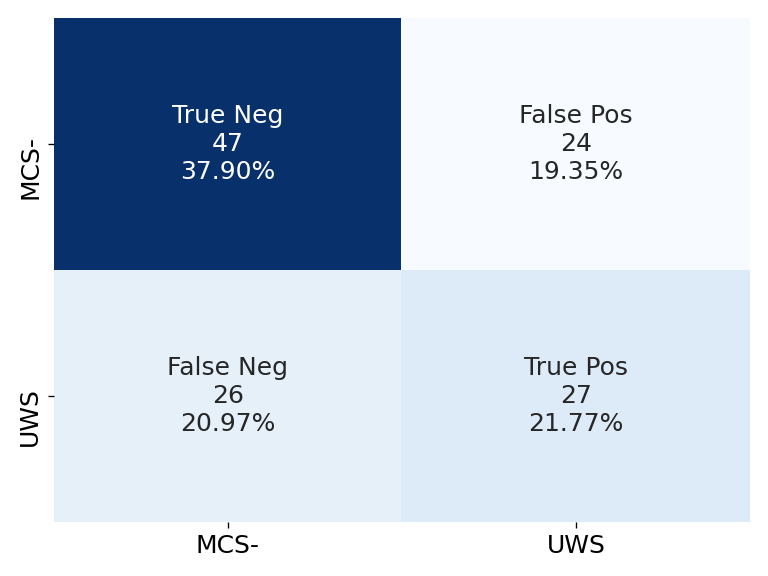


1. ICH, UWS/VS vs MCS-


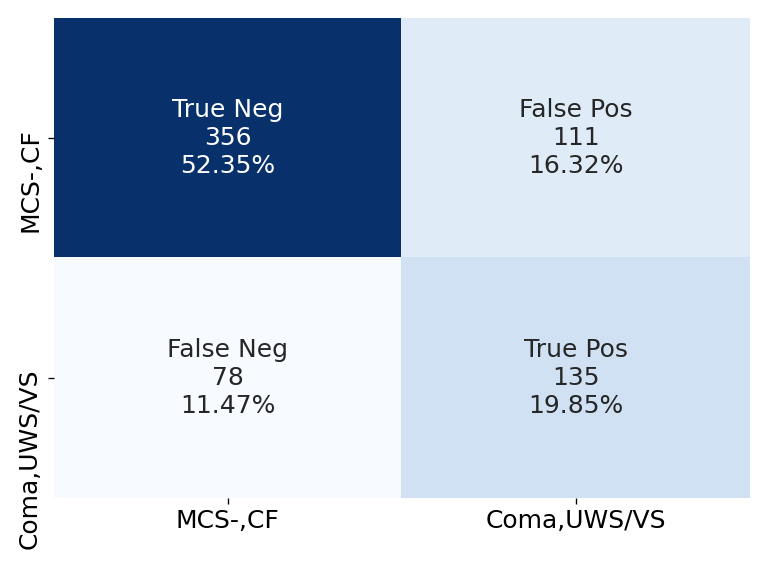

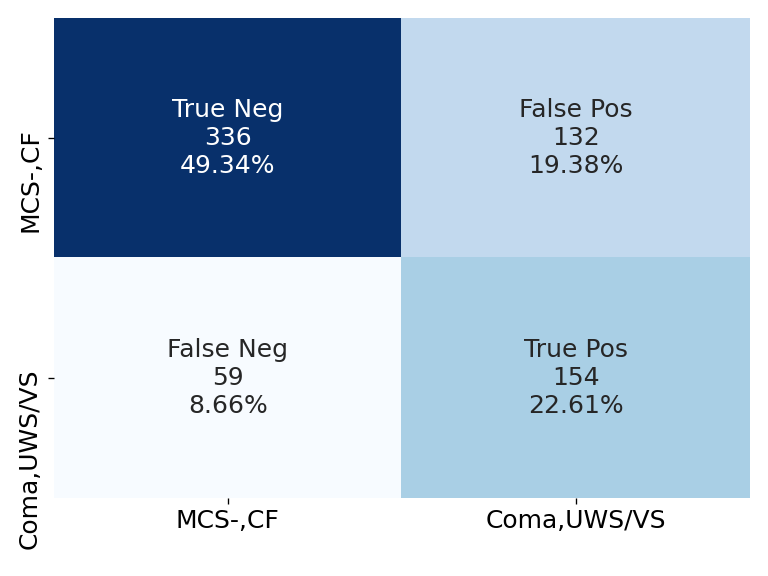


(B) ICH, Coma, UWS/VS vs MCS-, CF


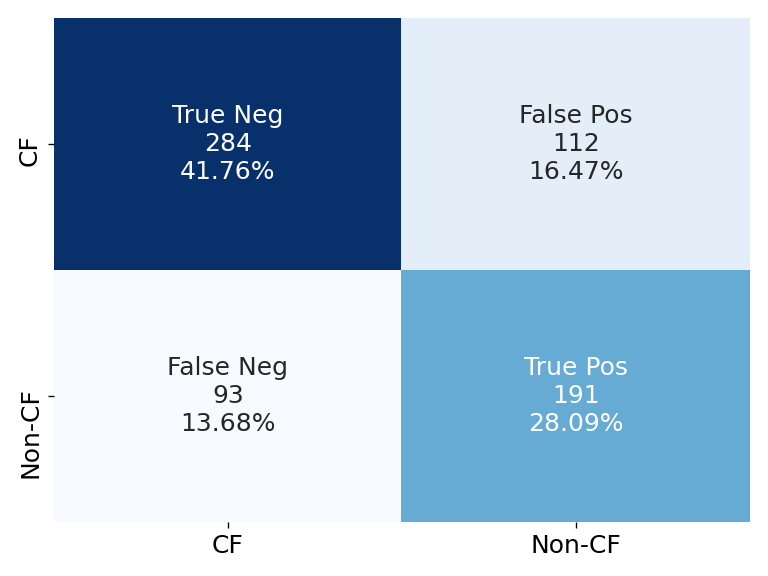

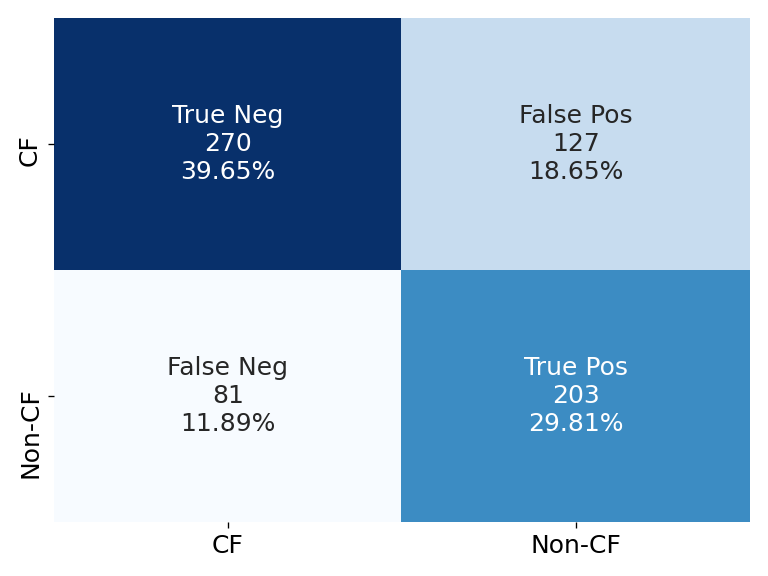


(C) ICH, Non-CF vs CF

**Predicted Label**

**True Label**

**Figure S3:** Confusion matrix showing performance for a classification task (from top to bottom) and physiological variable subsets (from left to right) on the ICH patient group for the LOPO framework. VS/UWS = vegetative state/unresponsive wakefulness syndrome, MCS- = minimally conscious state minus, CF = command following, and LOPO = leave one patient out, ICH = intracerebral hemorrhage, ICU = intensive care unit.
